# An overview of the bacterial microbiome of public transportation systems—risks, detection, and countermeasures

**DOI:** 10.3389/fpubh.2024.1367324

**Published:** 2024-03-11

**Authors:** Yen-Tran Ly, Stefan Leuko, Ralf Moeller

**Affiliations:** Department of Radiation Biology, Institute for Aerospace Medicine, German Aerospace Center, Cologne, Germany

**Keywords:** microbial community, public transport, microbial detection, countermeasures, microbial spread

## Abstract

When we humans travel, our microorganisms come along. These can be harmless but also pathogenic, and are spread by touching surfaces or breathing aerosols in the passenger cabins. As the pandemic with SARS-CoV-2 has shown, those environments display a risk for infection transmission. For a risk reduction, countermeasures such as wearing face masks and distancing were applied in many places, yet had a significant social impact. Nevertheless, the next pandemic will come and additional countermeasures that contribute to the risk reduction are needed to keep commuters safe and reduce the spread of microorganisms and pathogens, but also have as little impact as possible on the daily lives of commuters. This review describes the bacterial microbiome of subways around the world, which is mainly characterized by human-associated genera. We emphasize on healthcare-associated ESKAPE pathogens within public transport, introduce state-of-the art methods to detect common microbes and potential pathogens such as LAMP and next-generation sequencing. Further, we describe and discuss possible countermeasures that could be deployed in public transportation systems, as antimicrobial surfaces or air sterilization using plasma. Commuting in public transport can harbor risks of infection. Improving the safety of travelers can be achieved by effective detection methods, microbial reduction systems, but importantly by hand hygiene and common-sense hygiene guidelines.

## 1 Introduction and discussion

Viruses play a major role in the spread of infectious diseases, most recently SARS-CoV-2, which was responsible for the COVID-19 pandemic. Even before the occurrence of SARS-CoV-2, the Influenza waves are causing 15,000–70,000 deaths of European citizens every year (1).

However, in addition to viruses, bacteria are also responsible for the spread of infectious diseases. More than half of emerging infectious diseases are caused by bacteria, many of which are drug-resistant (2). Antimicrobial resistance has long been recognized as an acute danger and is also referred to in the literature as a silent pandemic (3). The spread of microorganisms and thus also pathogens does not necessarily begin in hospitals, but rather where people move around.

### 1.1 Humans represent the main source of bacteria within subways

Subway systems are widely used, especially in big cities and carry millions of passengers every day. The high frequency of passengers using public transportation facilitates an exchange of microorganisms, especially when getting in contact with frequently touched surfaces such as handrails, and sharing the air within a confined space. In this review, the most common taxa within the subway microbiome of different cities are presented, and relevant risk factors are discussed. Further, a range of microbial detection methods are listed and countermeasures that may be applied in public transport are described.

Touching objects, such as handrails, leads to a transfer of the human hand microbiome to the touched object. In recent studies, the transfer of the hand microbiome from test subjects to objects was demonstrated (4, 5), which can also be transferred to the subway environment. The most abundant organisms found in subway microbiome studies in various cities are displayed in Table 1. Among those, most frequently occurring taxa were *Acinetobacter, Staphylococcus, Propionibacterium, Corynebacterium, Micrococcus, Streptococcus,* and *Kocuria,* which are common for the human skin microbiome (15–17). These studies were not specifically focused on the detection of pathogens, and only a few were found such as *Helicobacter pylori*, *Acinetobacter* species (sp.) (10) as well as opportunistic pathogenic isolates related to the species *Propionibacterium acnes* and *Staphylococcus epidermidis* or genera *Pseudonocardia* and *Nesterenkonia* (14). An important factor that should be considered is that in most of the studies listed, microbial detection was based on 16S rRNA sequencing, which does not provide adequate detection at the species level (18, 19) and therefore, no pathogenic strains were conclusively detected.

**Table 1:** Overview of abundant bacteria across subways and subway stations found in multiple studies.

Organism			Location		Method	References
Five most abundant taxa (genus level): *Acinetobacter* *Corynebacterium* *Streptococcus* *Staphylococcus* *Cutibacterium*	Ubiquitous in all samples: *Acinetobacter* *Corynebacterium* *Streptococcus* *Staphylococcus* *Propionibacterium*	*Kocuria* *Pseudomonas* *Micrococcaceae* *Micrococcus*	Subway, Mexico city Turnstiles Stair handrails Escalator handrails Platform floor Train poles Seats		V3–V4 region of the 16S rRNA gene, MiSeq™ Illumina	(6)
Surfaces dominated by human skin and oral commensals: *Propionibacterium* *Corynebacterium* *Staphylococcus* *Streptococcus*			Subway, Boston Grips Poles Seats Seat backs	Touchscreens Sides of fare ticketing machines	V4 region of the 16S rRNA gene, MiSeq™ Illumina	(7)
Most abundant known genera: *Propionibacterium* *Corynebacterium* *Streptococcus* *Staphylococcus*			Metro, Mexico city Station turnstiles Vertical handrails inside the train		V3–V4 region of the 16S rRNA gene, MiSeq™ Illumina	(8)
Most commonly detected genera: *Micrococcus* *Enhydrobacter* *Propionibacterium*	*Staphylococcus* *Corynebacterium*		Subway/MTR (Mass Transit Railway), Hong Kong Aerosol samples		V4 region of the 16S rRNA gene, MiSeq™ Illumina	(9)
Bacterial species with the highest abundance: *Propionibacterium acnes* *Micrococcus luteus* *Propionibacterium humerusii* *Acinetobacter baumannii* *Staphylococcus epidermidis* *Escherichia coli* *Staphylococcus aureus*			Subway/MTR (Mass Transit Railway), Hong Kong Hands after handrail touching for 30 min		Metagenome sequencing with Illumina HiSeq™ 1,500	(10)
*Pseudomonas stutzeri* *Stenotrophomonas maltophilia* *Enterobacter cloacae* *Acinetobacter radioresistens* *Acinetobacter nosocomialis*	*Lysinibacillus sphaericus* *Enterococcus casseliflavus* *Brevundimonas diminuta* *Acinetobacter lwoffii* *Bacillus cereus*		Subway, NYC Turnstiles Emergency exits Metro card kiosks Benches Stairwell handrails	Trashcans Doors Poles Handrails Seats	HiSeq™ 2,500	(11)
High abundance: *Paracoccus* *Sphingomonas* *Kocuria* *Acinetobacter* *Staphylococcus*	Lower abundance: *Dietzia* *Streptococcus* *Enterobacter* *Enterococcus* *Anaerococcus*	*Blautia* *Burkholderia*	Metro, Athens Bioaerosol of underground station		16S rRNA gene and ITS, MiSeq™ Illumina	(12)
*Stenotrophomonas* *Pseudomonas* *Dietzia* *Brevundimonas* *Intrasporangiaceae_u*	*(Arsenicicoccus/unclassified)* *Comamonadaceae_u* *Staphylococcus* *Rhodococcus* *Erwinia*		Subway, Moscow Information stand Bench Floor	Wall Railings	V4 region of the 16S rRNA gene, MiSeq™ Illumina	(13)
Air: *Unassigned* *Micrococcus* *Staphylococcus* *Rubrobacter* *Sphingomonas* *Hymenobacter* *Arthrobacter* *Corynebacterium* *Nocardioides*	*Psychrobacter* *Blastococcus* *Kocuria* *Streptococcus* … Surface: Unassigned *Staphylococcus* *Sphingomonas*	*Streptococcus* *Hymenobacter* *Corynebacterium* *Arthrobacter* *Kocuria* *Micrococcus* *Psychrobacter* *Flavobacterium* …	Subway, Oslo Air and surface samples from 16 stations Across four seasons		V3–V4 region of the 16S rRNA gene, MiSeq™ Illumina	(14)

There have been studies that investigated the public transport microbiome within different cities. The most common method was the 16S rRNA sequencing, which provides taxa data of the genera found within samples more or less confidently. Interestingly, but not surprisingly, the genera of bacteria associated with humans are repeatedly listed as passengers leave their microbial footprints in the passenger cabins. These typically found bacterial genera may also be helpful in terms of suitable model organisms to study effective measures to reduce microbial load and pathogens in public transportation to reduce the transmission of infectious diseases.

For this section, we reviewed 30 research articles, including nine studies on the subway microbiome, 12 studies on the occurrence of ESKAPE pathogens in public transportation environments, and 7 studies on general information on the human skin microbiome, the association with surfaces and the detection of pathogenic species in general.

#### 1.1.1 ESKAPE pathogens—detected in public transport?

ESKAPE pathogens are the causative agents of most nosocomial infections worldwide. The abbreviation stands for the species *Escherichia coli*, *Staphylococcus aureus, Klebsiella pneumoniae, Acinetobacter baumannii, Pseudomonas aeruginosa*, and *Enterococcus faecium/faecalis*. Those organisms can be highly virulent and carry or transfer antibiotic resistances (20, 21).

Antibiotic resistance and the spread of multidrug-resistant bacteria (AMR) is a problem that was associated with about 4.9 million deaths worldwide in 2019 (22). However, the spread of these organisms does not occur in hospitals alone, but also in places with a high frequency of people, such as on public transportation. Notably, the following studies were focused on the detection of pathogenic species, mostly based on (selective) cultivation followed by PCR of pathogen-associated marker genes, e.g., the *mcr-1* gene for *E. coli* that mediates colistin resistance.

The most prominent ESKAPE species within the public transport studies is the (opportunistic) pathogen *Staphylococcus aureus*. In general, due to the natural passenger’s microbiome, the skin-associated species are highly abundant in busses and subways. In a bus, serving both hospital and community routes, methicillin-resistant *S. aureus* (MRSA) was found (community-associated SCCmec type IV and healthcare-associated SCCmec type II). Of the detected MRSA strains, 65% were multidrug resistant (23). Within this study, seats and seat rails were most contaminated. In subways, *S. aureus* containing the *mecA* gene was detected, alongside natural skin-associated species of *S. aureus* (11)*. mecA* is associated with methicillin-resistant *S. aureus* (MRSA) and nosocomial infections, but the study concludes no strong evidence for pathogenicity based on the obtained sequences. Other studies showed the prevalence of MRSA in public transport (24–27).

*Escherichia coli* with a multidrug resistance, including *mcr-1* which mediates colistin resistance, was found in public transportation in Guangzhou, China (28). Twenty-three isolates of 737 samples with bacterial growth were positive for *mcr-1*, most of them were resistant against ampicillin, cefotaxime, fosfomycin, and gentamicin.

For *Klebsiella pneumoniae*, there were less findings of drug resistant isolates. In the Beijing (China) subway environment, highly touched surfaces were sampled and from a total of 603 samples across 15 metro lines, 11 carbapenem-resistant *K. pneumoniae* isolates were detected (29).

*Enterobacter* species were also found in public transport studies. *E. faecium* was abundant throughout the subway in New York City, United States (11), and multidrug resistant *E. faecalis* was observed on shared bicycles in Chengdu, China (30).

No studies have been found on the occurrence of multidrug resistant *Acinetobacter baumannii* and *Pseudomonas aeruginosa* in public transport.

### 1.2 Microbial detection methods

There are a number of options for identifying bacteria. Classical biochemical or physiological methods such as microscopy are time-consuming and inefficient when it comes to examining a large number of samples and identifying the organism. Most studies reviewed within this work used the methodology of next-generation sequencing (1.2.1), which displays a modern and high-throughput detection approach, in contrast to cultivation on nutrient media. Latter allows the analysis of organisms that can grow under specific conditions. Several other methods exist, such as matrix-assisted laser desorption ionization coupled to time-of-flight mass spectrometry (MALDI-TOF MS) (31), or tandem mass spectrometry (19).

#### 1.2.1 Next-generation-sequencing

Nowadays, next-generation-sequencing (NGS) is the most used technology for sequencing. With this approach, high throughput analysis is possible and enables the identification of microbes within a high sample size in a cost-effective manner, generating high amounts of data (32, 33). There is a big variation within the NGS DNA sequencing technologies, varying in amplification method, sequencing chemistry, sequencing speed, etc. (32, 33). The most established NGS platform was created by Illumina, followed by Oxford Nanopore, which revolutionized the field by releasing a first portable nanopore sequencing device in 2014. Each technology differs in its output, advantages and limitations. With NGS, not only the microbial identity can be detected, also the diversity within or of all samples can be determined, outshining the limited information obtained by cultivation. The possibilities within NGS and the bioinformatical analysis are rapidly evolving more and more. Nevertheless, there are some shortcomings when it comes to profiling uncharacterized species in environmental microbiomes, as strain-level analyses are usually tested for human metagenomes and the tools are tailored to human metagenomes (34).

#### 1.2.2 Loop-mediated isothermal amplification

Metagenomics is a powerful tool to identify the microbiome of a sample. If specific organisms are to be screened for, such as potential pathogens, there are methods such as loop-mediated isothermal amplification (LAMP) that allow the targeted detection of species. LAMP is a fast, cost effective, and easy tool to detect specific organisms and requires only a few devices, while the evaluation occurs after 30 min.

Using marker genes, which differ for every organism, fast and detailed detection with a high specificity and sensitivity are possible (35). The potential of LAMP has already been established in relation to the detection of pathogens in the food industry (36). It is also useful for hospitals or in human high traffic environments to monitor microbial threats. During the SARS-CoV-2 pandemic, several publications showed the successful application of reverse transcription LAMP for this pathogen (37–39).

To this date and to our knowledge, there is no publication on the use of LAMP for pathogen detection in public transport as microbial monitoring measure. The detection of drug-resistant organisms is an important factor in monitoring the spread of pathogens and has yet to be implemented.

### 1.3 Countermeasures and feasibility in public transport

**Figure 1:**
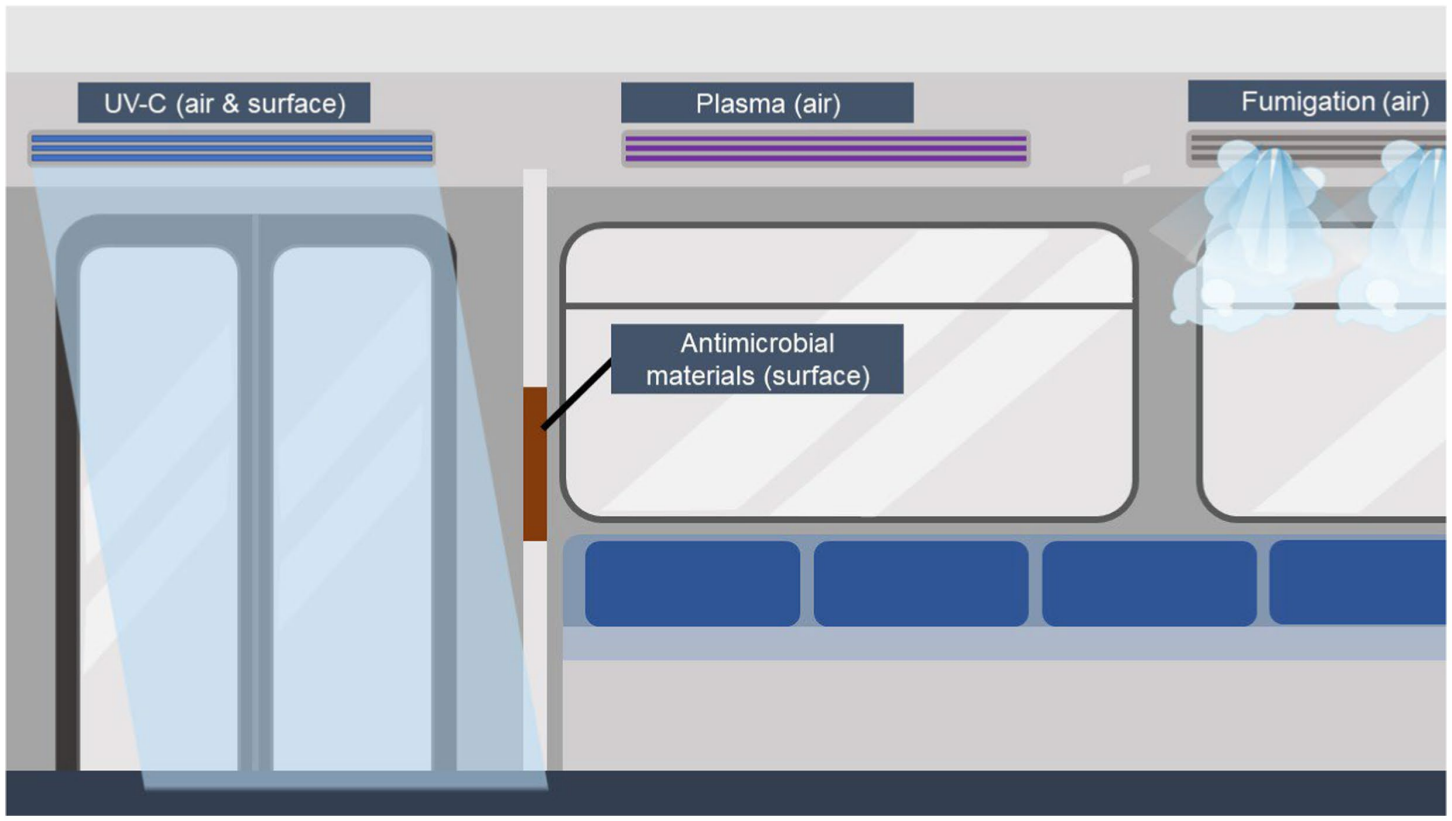
Overview of described microbial countermeasures in public transport. For air cleaning and disinfection, UV-C, fumigation of disinfectants, and plasma air sterilization can be used. Of those, plasma air cleaning is suitable for the use during passenger occurrence. For the reduced microbial burden on (highly) touched surfaces, antimicrobial surfaces can be implemented. UV-C can be installed during cleaning times without any passenger on board, as well as the fumigation of chemicals. Created with BioRender.com.

A summary of the mentioned countermeasures is displayed in Figure 1. The term antimicrobial includes not only bacteria, but also other groups such as viruses and fungi. But even within the group of bacteria, the effect of countermeasures varies depending on the bacterium, e.g., in the case of spore-forming bacteria, as their spores can be highly resistant to heat, for example (40, 41). Many of the mentioned countermeasures have been tested within hospital settings and in food industry, since the urge of clean and sterile environments is inevitable in those areas. Passenger cabins do not have to be sterile, but to provide an environment that does not promote the transmission of (opportunistic) pathogens, measures are needed.

#### 1.3.1 Antimicrobial surfaces

The transmission of pathogens is especially meaningful when it occurs through surfaces in epidemic and endemic scenarios (42). Although the transmission of pathogens through contact surfaces can be reduced by antimicrobial surfaces, the long-term usage and consequences have to be evaluated. One important factor is the increased risk of the development and transmission of antibiotic resistances between bacteria, when such materials are overused (43).

One of the best investigated antimicrobial material is copper. It causes cell damage by releasing copper ions which causes the cell membrane to rupture, leading to a membrane potential loss and depletion of cytoplasmic subtances (44). Further, copper ions induce reactive oxygen species (ROS), which in turn cause DNA damage (45). While copper as a material is costly, surfaces using the antibacterial effect of copper and integrating it as metal nanoparticles within a polymer matrix makes it cost effective, as reviewed by Tamayo et al. (46), and therefore could be suitable for a broad use.

While the antimicrobial properties of copper have long been known and researched, there are many different antimicrobial surfaces available (47, 48). For example, anti-biofouling surfaces can reduce microbial adhesion to the surfaces, biocidal nanocomposites kill microbes using biocidal species. Physical mechanisms as nanostructured surfaces can rupture bacterial cells, others can even prevent the attachment on the surfaces (49).

Some innovative antimicrobial materials were already tested in public transportation, such as antimicrobial photodynamic coatings, showing an absolute risk reduction of 22.6% for high bacterial counts (50). Other tested materials showed no significant reduction of the microbial burden, using photocatalyst-coated and uncoated hand-contact surfaces (51).

#### 1.3.2 Fumigation of chemicals as an antimicrobial approach

In the process of fumigation, an antimicrobial solution is nebulized in an enclosed environment with the aim to reduce the microbial burden in the air and on surfaces. The nebulization of chemicals, e.g., hydrogen peroxide has been in use (52, 53). There are also different forms of fumigation, that even consider the application in public transport (54). Here, peracetic acid stabilized with acetic acid and hydrogen peroxide showed an effectiveness of disinfection of 81.7% in busses, and even worked against highly resistant spores. Hydrogen peroxide facilitates the penetration of peracetic acid, which contributes to a fortified sporicidal activity of the agents, as tested with *Bacillus subtilis* spores (55).

The effectiveness of fumigation is highly dependent on the materials to be disinfected (54, 56), e.g., the effect of fogged peracetic acid and hydrogen peroxide was shown to be particularly high on glass windows and doors, and low on fabric materials (56). Further, the efficacy depends on the type of microorganism, the fumigation device and technology and the substance (57–59). One downfall of the fumigation of chemicals is the safety measures, that have to be ensured. Therefore, the usage of fumigation can only occur while the passenger cabins are not in service, but could be performed during night times. Considering the costs of fumigation, it depends on the device and fumigation technologies used. Costs for consumables are low, e.g., ~2 € / L of hydrogen peroxide.

#### 1.3.3 UV-C

Another method for disinfection in public transport, but more commonly employed in hospital settings, is UV-C disinfection. UV radiation causes DNA damage (60), which is mediated by the generation of ROS (61). UV-C operates in a spectrum of 200–280 nm. Because UV-C is also harmful to humans, some efforts have been made to employ mobile robots for disinfection with UV-C radiation (62, 63). In hospitals, there have been several systems using and testing UV-C disinfection, that are combined with disinfectant chemical agents (64, 65). One disadvantage of this approach is the material damage (66) and the incomplete light contact in all areas in a room or cabin. An advantage of UV-C disinfection is the economical aspect. Some low-cost UV-C light devices can be purchased with high efficacy against strains of *Candida auris*, MRSA, and bacteriophage Phi6 (67). Although UV-C disinfection shows effectiveness against some pathogens, it can cause bacterial mutations (68). A new, LED-based UV-irradiation technology has shown to be effective against some bacteria and viruses, but it is connected to high costs (69), which is uneconomical for use in public transport.

#### 1.3.4 Plasma sterilization

A tool designed to provide both air purification and surface disinfection is plasma. Plasma is also known as the fourth state of matter, which is a particle mix with a high electrical conductivity and is chemically reactive. The use of plasma is well established in the food industry (70) and in the medical field (71, 72), but it may be useful for the application in public transport.

The antimicrobial effect of plasma has been long known and is created by the combination or single effect of charged particles (ions, electrons), reactive species (e.g., ozone, ROS), radiation of UV-C/Vacuum-UV (VUV) as well as heating (73–75).

There are different types of plasma that can be used for disinfection. In a study conducted by Liang and Wu (76), culturable bacterial aerosol diversity loss was observed after using non-thermal plasma. Tested with *Aspergillus niger, Bacillus subtilis,* and *Pseudomonas fluorescens* as test organisms, it was described as a highly efficient air decontamination method.

To date, no study has used plasma as a system to reduce the microbial load in public transport. Only plasma related methods, such as a needle-point bipolar ionization system was tested in trams to investigate the reduction of bioaerosols (77). It was shown that environmental bioaerosols were reduced with this method, but it was not sufficient for surfaces. Therefore, more research is needed to test the feasibility of plasma technologies in the public transport context. Regarding cost-efficiency, only publications are available on the use of plasma in water treatment plants or in food industry (78), using plasma activated water (79). But in general, the formation of non-thermal plasma is connected to low energy input, unlike thermal plasma (80).

All approaches that were introduced in this section have their advantages and disadvantages. Different factors have to be considered when finding a best suiting method for a specific environment, such as passenger cabins. These include cost-effectiveness, service life, operation of devices, combined with the effectiveness of microbial reduction. In the end, the aim to apply countermeasures within the passenger cabins is to reduce the microbial load and therefore decrease the spread of potential pathogens, and a combination of some methods may bring all advantages together and ensure passenger safety and comfort.

## 2 Conclusion

In this review, the most common bacterial organisms from studies of the public transport microbiome were presented. Most studies performed 16S rRNA sequencing to identify the microbiome. The results showed that the public transport microbiome is dominated by human-associated organisms, while no pathogens were detected. However, targeted studies have shown that many of the so-called ESKAPE organisms in particular are found in public transportation and that this can be the place for the transmission of pathogens.

The use of the presented countermeasures in public transport was classified in this review. The purpose of this research is to show what is shaping our microbiome in public transportation and how specific organisms can be detected, but also reduced, to create a safe environment where pathogen transmission is minimized. However, this review also shows that more research is still needed to establish microbial reduction measures in public transportation.

## Author contributions

Y-TL: Conceptualization, Visualization, Writing – original draft, Writing – review & editing. SL: Conceptualization, Writing – review & editing. RM: Conceptualization, Funding acquisition, Supervision, Writing – review & editing.
